# From Kierkegaard to Langer (From Kierkegaard’s paradox to Langer’s psychology of possibility)

**DOI:** 10.3389/fpsyg.2014.01161

**Published:** 2014-10-16

**Authors:** Sayyed Mohsen Fatemi

**Affiliations:** Department of Psychology, Harvard UniversityCambridge, MA, USA

**Keywords:** psychological ontology and philosophical ontology, mindfulness, paradox, choice, existentialism

## Abstract

This paper presents the connection between Kierkegaard as a philosopher and Langer as a psychologist in opening up the possibility of dialogical interactions between philosophy and psychology. The paper demonstrates how both Kierkegaard and Langer transcend the pre-established parlance of the disciplinary paradigmatic analysis and invent new and interdependent multidisciplinary perspectives. Through numerous examples of their underlying viewpoints, the paper explores how the relation between psychological ontology and philosophical ontology may facilitate the process of a leap beyond epistemological propositions.

## INTRODUCTION

When psychology departed from philosophy and claimed a new realm of authority as a discipline it practically disengaged itself from ontological explorations. This, however, did not rule out the potential and ongoing dialogical indebtedness of each discipline. Some including William James in the past and some contemporary ones such as Paul Ricoeur demonstrated the dialogical interactive process of both disciplines.

Kierkegaard is a philosopher who gave rise to a new understanding of philosophy and propounded that philosophy was not to be circumscribed within abstract concepts but needed to open up an accessible touch with practical facets of life. He suggested that philosophy’s task would transpire in a thoughtful examination of real lived experiences. Instead of an indulgence in the previously common philosophical practice of abstract oriented concepts and categories, Kierkegaard embarked on bringing philosophy to the phenomenological realities of every one’s life and highlighted how everyone can be in dire need of philosophical reflections. In doing this, Kierkegaard did not abide by the sovereignty of reason as emphasized by the preceding philosophical schools but illustrated the power and the panacea of passion in changing and transforming minds and hearts.

Kierkegaard identified the greatest malaise of our time in the selflessness of people and elucidated how the dissipation of self has tightened the circles of meaning and contributed to the emergence of sundry psychological malfunction. He deplored the severe engrossment in the self-denying parade of quotidian utilitarian engagements and vociferously called for an authentic return to the sphere of the self where the real security could be found. Kierkegaard considered inwardness as the panacea of recuperating from the barrage of meaninglessness and explicated the itinerant of the self as the one who would manage to experience the composure and consummation. He suggested that the real sense of aesthetics would unfold itself not in the exterior cynosures of beauty but in the internal avenues of revitalization where the celebration of the self would be attuned to emergent understanding of the process of life and its associative contextual becoming.

Kierkegaard demonstrated how knowing could be pretentiously entrapping where one would be incarcerated in the dungeon of his/her mindset and assumptions. He elucidated how an unassuming life would be much more liberating than an overwhelming ripple through the unbreakable walls of knowing. Kierkegaard described every one’s unique involvement in filling the jar of life as a great phase of responsibility as he expounded on the necessity of passion for achieving one’s authenticity.

Kierkegaard highlights the significance of choice for everyone and argues that people may not experience being free if they don’t experience the power of choice in their life namely, if they do not choose themselves. Choice is a stage that people need to be constantly aware of. It is through finding a direction or a purpose in one’s life that one can experience real freedom. Kierkegaard removes the stability of essence and nature for human beings and underscores the power of choice in transforming what one can be and wants to be.

In line with an existential move that calls for moving forward with the blessing of choice, a name in the discipline of psychology appears to delineate numerous common denominators with Kierkegaard. Langer is known as the psychologist who has profusely depicted the grace of mindfulness and its implications in interpersonal and intrapersonal life for the past thirty five years. Her findings in the field of psychology have instantiated how our life can be devoid of any presence when we experience mindlessness. Langer, through dozens of psychological experiments, has elaborated how mindlessness imposes paralysis of action and gets one stuck in his/her position of assumptions. She indicates how a self without mindfulness would lose his/her potentials as he/she would drift from the process of becoming.

Langer discusses how the power of one’s choice would promise the renaissance of self as one openly experiences the horizons of becoming. In doing so, Langer argues, one needs to constantly pinpoint being mindful in the midst of a wide variety of mindless- inducing prompts. She demonstrates how openness toward the spectrum and perspectives may help one explore the possibility of understanding different angles and facets that may be neglected when one is entrapped in mindlessness.

Langer’s self-renaissance project is tied to the transformative power of language where the stamina of dictions and words are shown to develop various formative beings. She argues how a constrained approach toward the horizons of possibilities would deprive one of welcoming new information. Langer describes the tyranny of the seemingly helpful cognitive schemas as one of the main contributors of mindlessness. She propounds a sagacious sensitivity toward context where things, events, people, and phenomena can be understood within their contextual terms. An approach devoid of contextual understanding would lead to marginalization of some attributes or negation of otherwise salient features.

Langer depicts the liabilities of knowing and its objective gestures while appreciating the position of not knowing. She underlines the significance of emancipation from the intensity of assumptions and proposes a mindful search for the innovative leaps when one is persistently urged to remain in the familiar reference points. Langer’s mindfulness is revolutionary in that it calls for novel experiences of being. She does not consider the transformation of cognitions sufficient for the commencement of authentic changes but she calls for radical transformation of consciousness.

Langer explicates how our era’s mindlessness has provoked counterproductive intra personal self talks, misleading health projects, nefarious intercultural models and deleterious educational policies. She argues that most of our learning takes place mindlessly and thus we don’t experience the presence of learning.

Langer identifies how mindlessness would solidify the severity of judgmental attitudes whereas mindfulness expands the realm of comfort. Stress is induced in the vicious circles of mindlessness while tranquility is poured out in the presence of mindfulness.

Langer considers attention toward the perspective of the actor as the necessary component of understanding one’s core concerns and critiques the psychological approaches that prescribe the sufficiency of the observer’s perspective in representing the psychological reality. Langer’s mindfulness is a critique of the positivist psychology and its reductionist claims. She pinpoints how labels and words and their extensive cultivation and socialization can foster mindlessness and weaken people’s understanding of their choices. She displays examples and cases where mindlessness has lead to paralysis of action. She recounts the socially constructed certainty of assumptions and their impeding implications. She argues that we are constrained by the subjugation of our recursive patterns of our mindlessly accepted mindsets where possibilities are narrowly defined.

## KIERKEGAARD AND LANGER

Kierkegaard’s standpoint on philosophy is uniquely interwoven in the play of passion and practice. He disdains the view point on philosophy that is merely engaged in the abstract conversations of the past. In his pseudonymous book “either/or,” Kierkegaard (1959) demonstrates how his philosophy is not in pursuit of the same principles of his contemporary philosophers and indicates that

The philosopher says, “That’s the way it has been hitherto.” I ask, “what am I to do if I don’t want to become a philosopher?” For if I want to do that, I see clearly enough that I, like the other philosophers, shall soon get to the point of mediating the past… There is no answer to my questions of what I ought to do, for if I was the most gifted philosophical mind that ever lived in the world, there must be one more thing I have to do besides sitting and contemplating the past” p.175 (171).

Langer’s psychology also begins with a departure from the inadequacies of the pervasive psychological reductionist discourses and the salience of “knowing what is” and its past oriented predilections. Instead, it focuses on “what can be” being very similar to numerous notions presented by Kierkegaard including the idea of forwarding, choice and inwardness. Langer speaks the language of positivist psychology to corroborate the inadequacy of the empirical psychology. She questions the sovereignty of the empiricism and indicates how the entrapment within the empirically established propositions may lead to acting from a single perspective. Langer (2009) illustrates the implications of questioning the determinacy of “is” for psychological certainty and indicates that

my research has shown how using a different word, offering a small choice, or making a subtle change in the physical environment can improve our health and well-being. Small changes can make large differences, so we should open ourselves to the impossible and embrace a psychology of possibility, p. 15.

Langer’s psychology of possibility may be discussed in line with Kierkegaard’s discussion of subjectivity and objectivity where Kierkegaard presents subjective truth in relation to an ontological way of living where one lives in truth or truth becomes a way of being as a human being. He distinguishes subjective truth from objective truth where truth is subsumed under one’s category of knowing.

In Concluding Unscientific Postscript, Kierkegaard (1992) asserts that

objectively the emphasis is on what is said; subjectively the emphasis is on how it is said… But this is not to be understood as manner, modulation of voice, oral delivery, etc., but it is to be understood as the relation of the existing person, in his very existence, to what is said. Objectively, the question is only about categories of thought; subjectively, about inwardness…Only in subjectivity is there decision, whereas wanting to become objective is untruth. The passion of the infinite, not its content, is the deciding factor, for its content is precisely itself. In this way the subjective “how” and subjectivity are the truth, p. 203.

Langer’s psychology of possibility calls for Kierkegaard’s subjective truth of possibility in that the possibility needs to be ontologically experienced so the phenomenological experience can testify the truth of the experience. For the same reason, Langer’s (2009, p.18) psychology of possibility targets the mindsets and their stability as the commencement of exploring the possibility of change as she iterates that “we imagine the stability of our mindsets to be the stability of the underlying phenomena and so we don’t think to consider the alternatives.” Langer’s focus on possibility goes beyond the epistemological possibility and highlights the significance of ontological possibility. The roadblocks, Langer argues, to understanding ontological possibility happens in the realm of epistemology where the commitment to specific epistemological propositions hamper exploring the avenues of new possibilities. Langer focuses on the paralyzing power of cognitions when they tend to stabilize their certitude in view of their frequency, their repetition, their cultivation, and their socialization. Langer indicates how possibility can be limited and limiting when one is forcibly circumscribed in the prescriptive and proscriptive modes of possibility. The leap from the limiting sense of possibility to the liberating sense of possibility, according to Langer, begins with questioning the province of possibility, namely the mindsets that describe the realm of possibility. Through her experiments, Langer questions the borders of possibility and revisits the quantifiers of propositional possibilities in which certain quantifiers are known to apply for acknowledging certain possibilities. Langer peruses Kierkegaard to elucidate the possibility of transcending the realm of established possibilities.

Kierkegaard discusses possibility as a unique and distinctly human feature which can transpire in different levels of existence and bring about different ways of living. Human beings are the only creatures, in Kierkegaard’s view point on possibility, which can go beyond the biologically established configuration and experience possibilities. Each realm of existence unfolds a different aspect of possibility.

The aesthete is indulged in the spectrum of possibilities and playfully experiences possibilities as he/she experiences the joy and pleasures of life whether physical beauty or intellectual enjoyments. In this stage of being and possibility, the aesthete is merely overwhelmed by a hedonistic predisposition toward possibilities and his/her life would be devoid of any meaning. His/her freedom is ontologically concealed to oblivion to the effect that he/she solely thinks of possibilities and their infinite, unconstrained, and unlimited expansion.

In the ethical stage of possibilities, the ethical person takes responsibility and acknowledges the power of freedom and decision making. The ethical person, according to Kierkegaard, understands the limitations of possibilities as he/she experiences a moral way of living. This stage of possibility can be induced by either an inner or external sense of values. It can be epitomized in one’s obedience to his/her own inner voice, in the exterior manifestations of law abiding attitudes or in the accumulative plethora of prescriptive codes, conventions and values. As the person experiences this realm of possibility namely the ethical domain, he/she experiences his/her action, decision making and freedom and thus he/she ascertains the power of responsibility to proceed with an action. Furthermore, he/she learns to be more in touch with his/her inner self as he/she experiences the undertaking of a moral action. On the other hand, the ethical realm of possibility highlights the limitations and constrictions that are associated with a moral undertaking as one, for instance, accepts the limitations that are interwoven with having a job. The ethical sphere, as a level of possibility, may lead to despondency and despair as it is embedded within a vulnerable system that is subjected to failure. It is intrinsically subjected to human errors.

The third level of possibility, according to Kierkegaard, happens in the religious sphere where passion, inwardness, truth, fullness, and power demonstrate themselves. The religious stage is a stage where one is deeply engaged with the inner life a possibility which does not occur for the aesthete nor for the ethical. The religious realm of possibility characterizes the most passionate mode of human possibility. The religious sphere of possibility portrays the relationship with God and acknowledgment of eternity. It illustrates the infinite possibilities and its widening horizons. The religious sphere reveals the profound layer of spirituality where one practically experiences being an itinerant of the inner life with passion and faith. The religious sphere is imbued in the personal testimony of the presence of God. The religious sphere relates one to one’s infinite potentiality. According to Kierkegaard, the religious sphere provides the possibility where becoming a self becomes possible where one needs to be related to oneself and more important than that one needs to be related to God that is the power that creates and constitutes the self.

It is important to note that these levels of possibilities, in Kierkegaard’s view point, are not explicating the ways of believing or knowing and they are not describing the cognitive and epistemological framework of someone but they are presenting how one is living thus they are ontologically defining one’s stages of being.

This may lead us to explore the role of self in Langer’s (2005, p. 21) psychology of possibility where she discusses how “a self that is absorbed in itself may be a self that is cut off from itself.” Langer’s portrayal of such a self demonstrates an implicit link to Kierkegaard’s spheres of possibilities where the experience of self can be stopped when there is no connection to the sphere of possibilities where the self can be fostered or revitalized. Langer (2005, p. 21) implicates this when she indicates that “my work has led me to conclude that he loneliness, boredom, and feelings of inadequacy people experience are usually the results of a lack of connection with themselves.”

Kierkegaard’s aesthete sphere of possibility can be of great significance in clarifying Langer’s notion of a self that is disconnected to itself. The aesthete lacks a self since he/she is devoid of an experience of a choice, of awareness of a commitment and of a decision to make.

Self, in Kierkegaard’s perspective, is what one does, of what one undertakes. It is about accomplishing a task. We may see this notion of accomplishment of a task in Heidegger’s concept of “authenticity” as distinct from “inauthenticity” where the self unfolds itself through the choices one makes or fails to make and thus loses its authenticity. Langer opens up the possibility of understanding how choices can elevate the sense of self as they highlight the observational role of self in creating, managing, and reinventing numerous choices.

It seems that Langer (2005, p. 27) is serving as heir of Kierkegaard when she discusses the role of choices for the self and suggests that “that is the essence of a personal renaissance, to learn to act and engage with ourselves mindfully, creatively, actively, and happily.”

Contrary to the atheistic existentialism of Sartre and Camus, Kierkegaard considers Langerian renaissance of self through a connection to spirit as he elucidates that

a human being is a spirit. But what is spirit? Spirit is the self. But what is the self? The self is a relation that relates itself to itself or is the relation’s relating itself to itself in the relation; the self is not the relation but is the relation’s relating itself to itself. A human being is a synthesis of the infinite and the finite, of the temporal and the eternal, of freedom and necessity, in short, a synthesis. A synthesis is a relation between two. Considered in this way, a human being is still not a self (Kierkegaard, 1989, p. 43).

Kierkegaard laments against the selflessness of the modern age and deplores the impediments that get the in way of people’s actualization of the self. Kierkegaard argues that dissipation of self contributes to our despair and despondency as it hampers our being fully human. Similar to Kierkegaard’s attack on the modern age selflessness, Langer’s psychology of possibility rails against the mindlessness of our age and rages against its implications in a wide variety of domains. Langer (1975, 2009; Langer et al., 1985) argues that mindlessness curtails our possibilities as it works against our creativity. Mindlessness, according to Langer, paralyzes our power of choices and imposes passive and automatic behaviors. Through our mindlessness, we depreciate the value of our being a human as we lose our sense of control over what we do and how we do. In line with Kierkegaard and Heidegger, Langer uses the term authentic selves and considers mindlessness as the roadblocks that prevent us from experiencing the genuine and authentic self.

Langer’s use of possibility bears close resemblance to what Aristotle pointed out in his *Poetics,* that the function of poetry is to represent what might be, rather than what has been. Kierkegaard presents possibility in the same line in his early writings and demonstrates how possibilities are going to be infinite in the realm of spirituality and the impossibility of possibility can fade away when the inwardness opens up the room for multitudes of possibilities, albeit impossible in a world governed by reason and analytical reasoning. Kierkegaard’s critique of Hegelian philosophy and its focus on rationalism propounds the significance of passion instead of reason with passion opening up the avenues of possibilities. The power of passion and its creational possibilities is characterized in the internal quest for spiritual connectedness and inwardness as Kierkegaard exemplifies Abraham as the hero of faith and possibilities.

Langer conducts a similar critique of the positivist psychology and its authoritative claims for owning the truth. Langer’s psychology of possibility enumerates the failures and flaws of the positivist driven psychology and elaborates how mindless driven psychology can turn out to be imposing in predictions and assessments. In stipulating the ramification of the critique against the positivist system, Langer (1997) argues that

the very notion of intelligence may be clouded by a myth: the belief that being intelligent means knowing what is out there. Many theories of intelligence assume that there is an absolute reality out there, and the more intelligent the person, the greater his or her awareness of this reality. Great intelligence, in this view, implies an optimal fit between individual and environment. An alternative view, which is at the base of mindful research, is that individuals may always define their relation to their environment in several ways, essentially creating the reality that is out there. What is out there is shaped by how we view it, p. 100.

Langer’s thirty five year long research discloses the price that we have paid for the tyrannical mindlessness of the positivist psychology and its unquestionable interventions in defining what is true. Her critique of the objectivity depicts the implications of our deep-seated submission to the ruled-governed world of scientism and indicates how the objective laden psychology has failed to explore the contexts and their role in meaning making. Langer (1989, 2009) discusses how the position of knowing in the framework of objectivity has ignored realities of contexts in sundry facets of human life. Langer (2005) suggests that we should be better off if we proceed with the position of not knowing and indicates that

Science, which prides itself on its objectivity, usually hides its choices from us even as it reports its findings. Many design choices that go into even our most rigorous scientific studies affect their outcomes. Greater awareness of these choices would make the findings less absolute and more useful to us. In fact, scientific research is reported in journals as probability statements, although textbooks, and popular magazines often report the same results as absolute facts. This change is done to make the science easier for the nonscientists to understand. But what it does, instead, is deceive us by promoting an illusion of stability. That illusion is fostered by taking people out of the equation-what choices the researcher made in sitting up the experiment, on whom it was tested, and under what circumstances. (p. 106)

On the implications of the dominant Western perspective in psychology, Wessells (1999), indicates that

In emergency situations, psychologists hired by NGOs or UN agencies often play a lead role in defining the situation, identifying the psychological dimensions of the problems, and suggesting interventions. Viewed as experts, they tacitly carry the imprimatur of Western science and Western psychology, regarded globally as embodying the highest standards of research, education, training, and practice. Unfortunately, the dynamics of the situation invite a tyranny of Western expertise. The multitude of problems involved usually stems not from any conspiracy or conscious intent but rather from hidden power dynamics and the tacit assumption that Western knowledge trumps local knowledge (pp. 274–275).

Langer’s emphasis on psychology’s epistemological crises of objectivity and its de-humanizing implications seems to establish her being an heir to Kierkegaard. Kierkegaard’s challenge of Hegelian rationality and the objectivity of Hegelians such as Martensen calls for revamping the foundations of knowing and knowledge as it does reveal the circumscribing pillars of objectivity in the discourse of rationality. In Concluding Unscientific Postscript, Kierkegaard’s pseudonymous Johannes Climacus argues that objectivity cannot give rise to inwardness. Kierkegaard claims that just as lack of objective truth can lead to madness, the “absence of inwardness is madness” too. Climacus illustrates a patient who has just escaped from a mental hospital and is worried about his recognition. He is worried that right after recognition, he will be sent back to the hospital so he thinks to himself:

“What you need to do, then, is to convince everyone completely, by the objective truth of what you say, that all is well as far as your sanity is concerned.” As he is walking along and pondering this, he sees a skittle ball lying on the ground. He picks it up and puts it in the tail of his coat. At every step he takes, this ball bumps him, if you please, on his bottom, and every time it bumps him he says, “Boom! The earth is round!” He arrives in the capital city and immediately visits one of his friends. He wants to convince him that he is not crazy and therefore walks back and forth, saying continually “Boom! The earth is round!” p. 195.

Kierkegaard considers the phenomenological perspicacity and wisdom of one’s life in one’s mindful engagement and awareness of his/her life. The madman in the example corroborates his insanity although he highlights the objectivity of a truth that is ineluctably unquestionable for everyone. The madman’s objectivity, however, is devoid of any reflexivity namely he cannot reflect on himself. Logical positivism in Langer’s perspective and logical calculations in Kiekegaard’s view point are similar to the mad man in Kierkegaard’s above mentioned example in that they both enunciate the truth while being mindless about the context in which the truth is embedded.

For Langer, mainstream psychology has been mainly obsessed with the legitimacy of the observer’s perspective known as the expert’s perspective and has marginalized and neglected the actor’s perspective. The legitimacy of the expert’s perspective, according to Langer, is largely due to psychology’s ownership of objectivity. The possession of objectivity and its accessibility for positivist psychology is explained by virtue of the rigorous methodologies implemented in psychology. Langer’s critique of the monopoly of the perspective in the eyes of the observer namely the expert produces sundry implications for numerous domains of human psychology. Langer (1975, 2009) claims that the actor’s perspective can open up a new world of possibilities a world which can be easily concealed to oblivion through the hegemony of the observer’s perspective.

Kierkegaard (1992) explicated the importance of mindfulness toward the actor’s perspective in a wide variety of contexts. He demonstrates how the superiority within the observer’s perspective can be detrimental in communicating the truth and suggests that

Take the case of a man who is passionately angry, and let us assume that he is really in the wrong. Unless you can begin with him by making it seem that it were he who had to instruct you, and unless you can do it in such a way that the angry man, who was too impatient to listen to a word of yours, is glad to discover in you a complacent and attentive listener-if you cannot do that, you cannot help him at all. Or take the case of a lover who has been unhappy in love, and suppose that the way he yields to his passions is really unreasonable, impious, unchristian. In case you cannot begin with him in such a way that he finds genuine relief in talking to you about his suffering and is able to enrich his mind with the poetical interpretations you suggest for it, notwithstanding you have no share in this passion and want it to free him from it-if you cannot do that, then you cannot help him at all; he shuts himself away from you, he retires within himself…and then you just talk at him. (p. 45).

Langer’s presentation of mindfulness can be elucidated with a profound understanding of the process of inwardness in Kierkegaard’s view point. Kierkegaard, as mentioned earlier, contends that the biggest ailment and pathology of the modern age can be attributed to selflessness. Heidegger (1995) speaks of such selflessness when he discusses “self-forming emptiness” (p. 126). Heidegger (1995, 127) suggests that our mindlessness about the emptiness of the self is so pervasive that the emptiness becomes “peculiarly inconspicuous.” In other words, we don’t see its obviousness as we are subjected to the mindlessness of the emptiness obviousness.

Cushman (1990), reiterates the ramifications and corollaries of mindlessness toward self-emptiness and selflessness when he indicates that a selfless person and an empty self is the one

[who] seeks the experience of being continually filled up by consuming goods, calories, experiences, politicians, romantic partners, and empathic therapists in an attempt to combat the growing alienation and fragmentation of its era. [It] is dependent on the continual consumption of nonessential and quickly obsolete items or experiences. accomplished through the dual creation of easy credit and a gnawing sense of emptiness in the self (p. 601).

Langer’s discussion of the implications of mindfulness (p. 24) also suggests that with an increase of a creative mindfulness, one can get proactively engaged in a search for “the inviolable self.” Langer (1975, 2009; Langer et al., 1985) develops a relationship between mindlessness and being scattered in multiple domains of self-negating pretentiousness. Our mindlessness is rearing absence from our fullness as it detaches us from our self-reflection. Mindlessness, in this sense, is a state of being fraught with gigantic engagements of Kierkegaardian selflessness whereas mindfulness presents a fundamentally different form of being in which one is connected to self.

Kierkegaard uses ‘paradox “to suggest a language and a sphere that is not comprehensible through the conventional reasoning. When one faces walls, Kierkegaard suggests, one is forced to accept the limitation and attests to the impossibility of going beyond the walls. The language of faith, love and prayer would open possibilities according to Kierkegaard as it supersedes the paradigmatic circumscription and offers novel horizons of exploration. This looks like absurd as it is not translated through the previously accepted language of the identified mindsets and it serves as a paradox since it stands against the ratiocination in the context of cognitively composed schemas.

Langer discusses the psychology of possibility in line with the same spirit as she removes the limiting layers that prescribe certain modes of thinking. Langer considers mindfulness as the key to creativity and novelty (Langer, 2009). In promoting the language of Kierkegaardian Paradox, Langer concentrates on the ineluctability of identified schemas in the domain of human cognition for understanding new categories and argues that the paradigmatic and syntagmatic implications of the identified categories would prescribe specific contemplative predispositions with special commitments.

The range and magnitude of the identified schemas both in concepts and propositions would suggest both descriptive and prescriptive moves that ultimately present a stable version for the so called reality. The reality is thus understandable within the sphere of the pre-identified commitments. Through a focus on the search for noticing the infinite flow of novelty, Langer invites the observer to fight for “otherwise” in the midst of the familiar (Fatemi, 2009).

Langer (1997) becomes united with Kierkegaard as she highlights the essence of effective teaching in the process of a disengagement from solipsism. A mindful teacher, according to Langer, is the one who constantly questions the position of knowing and tries to look into the perspective of the learner not only from the cognitive but the social and emotional perspective. This understanding of the context helps both educators and learners to become prepared for understanding their reference points in a broader perspective. It seems that Langer has been proactively inspired by what Kierkegaard when he indicated that

No, to be a teacher in the right sense is to be a learner. Instruction begins when you, the teacher, learn from the learner, put yourself in his place so that you may understand what he understands and in the way he understands it…. (Kierkegaard in Bretall, 1946, p. 335).

It is interesting to see that both Kierkegaard and Langer use narratives to present their psychological and philosophical demonstrations. Kierkegaard tells the story of a lily “more beautifully clothed than Solomon in all his glory,” who was “joyful and free of care all the day long” (Kierkegaard, 1998a,b). The lily, Kierkegaard narrates, is influenced by a “naughty little bird” that induces comparison and reports on the beautiful flowers in other places where the birds come up with the best songs ever. The lily begins to loathe itself and thus allows the bird to take it away to those glorious places. The lily is thus detached from the soil it belongs to and goes with the bird. On the way, the lily perishes and dies. According to Kierkegaard, the lily is the demonstration of human beings and the little naughty bird represents the comparison that entangles human beings. The soil also displays the roots and connectedness. Langer presents experiments and cases that indicate how the malaise of comparison may prevent one from exploring the genuine and authentic modes of living and expressiveness. Langer (2009) demonstrates how an entanglement in the comparison oriented attitude would impose a detachment from process and would ignore the authenticity of one’s province of choices. Authenticity, Langer argues, unfolds itself in light of discovering the pearl of choices and that happens in the apex of mindfulness.

## Conflict of Interest Statement

The author declares that the research was conducted in the absence of any commercial or financial relationships that could be construed as a potential conflict of interest.
